# Enhancing the Chemosensitivity of MKN-45 Gastric Cancer Cells to Docetaxel via *B7H6* Suppression: A Novel Therapeutic Strategy

**DOI:** 10.3390/life14121546

**Published:** 2024-11-26

**Authors:** Elif Sibel Aslan, Nermin Akcali, Cuneyd Yavas, Sajjad Eslamkhah, Savas Gur, Lutfiye Karcioglu Batur

**Affiliations:** 1 Faculty of Engineering and Natural Sciences, Department of Molecular Biology and Genetics, Biruni University, Istanbul 34015, Türkiye; nakcali@biruni.edu.tr (N.A.); cyavas@biruni.edu.tr (C.Y.); mtaghizad@biruni.edu.tr (S.E.); 2 Biruni University Research Center (B@MER), Biruni University, Istanbul 34015, Türkiye; 3 Internal Medicine Specialist, Private Clinic, Çanakkale 17100, Türkiye; drsavasgur@gmail.com

**Keywords:** gastric cancer, docetaxel, *B7H6* suppression, chemosensitivity, siRNA-mRNA

## Abstract

Purpose: Although chemotherapy is one of the standard treatments for gastric cancer, the disease’s resistance mechanisms continue to limit the survival rates. *B7H6* (*NCR3LG1*), an immune checkpoint belonging to the B7 family, is significantly overexpressed in gastric cancer. This work investigated the possibility of using *B7H6* suppression to improve the effectiveness of the widely used chemotherapy medication docetaxel. Materials and Methods: In this study, MKN-45 gastric cancer cells were transfected for 24 h with siRNA targeting *B7H6*, and then, docetaxel was added at optimal inhibitory doses (IC25 and IC50). To assess the impact of this combination therapy, cellular viability, proliferation, and migration were assessed using MTT assay, colony-forming unit assay, and wound-healing assay, respectively. Additionally, apoptosis and cell cycle status were evaluated by flow cytometry. Moreover, using qRT-PCR, the gene expression of *B7H6* and indicators associated with apoptosis was also examined. Results: The sensitivity of MKN-45 cells to docetaxel was greatly increased by the siRNA-mediated knockdown of *B7H6*, resulting in a decrease in the drug’s IC50 value. When compared to each therapy alone, the combination of *B7H6* siRNA plus docetaxel at IC50 levels exhibited a significant increase in apoptosis rate. The volume of cells arrested at the sub-G1 and G2-M phase was shown to rise when *B7H6* siRNA transfection was combined with docetaxel. Furthermore, the combination treatment significantly decreased the ability of cells to migrate and form colonies. Conclusions: *B7H6* suppression increases the susceptibility of MKN-45 gastric cancer cells to docetaxel treatment, resulting in decreased cellular proliferation and increased rates of apoptosis. The present work underscores the possibility of enhancing treatment results in gastric cancer by merging conventional chemotherapy with gene-silencing approaches.

## 1. Introduction

According to GLOBOCAN estimates in 2022, 20 million new cases of cancer were diagnosed. Gastric cancer was the fifth most frequently diagnosed cancer, responsible for almost 4.9% of cancer cases and 6.8% of cancer deaths [1]. Gastric cancer is a highly malignant condition of the stomach, and while there has been a general decline in its occurrence over the last ten years, it remains among the most lethal illnesses. This cancer is often diagnosed at an advanced stage, and the primary course of treatment is first surgery followed by chemotherapy. Regrettably, the prognosis for patients post diagnosis is quite poor. Consequently, there is an apparent need for essential research on prevention and the discovery of novel therapeutic modalities [2]. Additional treatment modalities that are sometimes recommended after surgical intervention and chemotherapy include radiation, immunotherapy, and targeted therapy [3].

One strategy of interest in gene-silencing therapy research is the use of synthetic small interfering RNAs (siRNAs) molecules, which are created in labs to silence disease-related genes. Currently, siRNA molecules are being tested in clinical trials phases 1 to 3, with the objective of treating infectious illnesses and cancer [4]. siRNAs are a class of short (22–23 bp) non-coding, double-stranded RNA molecules, which are posttranscriptional gene silencers that bind complementary to the target mRNA, thereby preventing translation and the production of the target protein. An increasingly prominent and well-researched approach is the suppression of genes associated with cancer. This approach has the benefits of specificity, high power, and high efficiency [5,6,7].

Recently, an exciting new era in cancer treatment has begun as scientists and doctors work to find ways to circumvent treatment by harnessing the power of the immune system. The field has recently identified immune checkpoints as critical targets due to their regulatory mechanisms that impact immune responses [8,9,10]. The B7 family of immune checkpoints exerts a substantial influence on the regulation of T-cell activity within the immune system, either through direct or indirect means. The development of a new treatment method with high potential arises from the ability of cancer cells in human tumors to evade destruction by the immune system. This evasion is caused by an imbalance in the expression of immune checkpoints, which is a characteristic of these tumors [11]. Human *B7H6* protein is not expressed in normal tissues; however, its expression has been detected in a number of malignancies. Preclinical findings reveal that *B7H6* might be a viable novel target for cancer immunotherapy [12].

Gastric cancer remains one of the most challenging malignancies due to its high mortality rate and frequent resistance to conventional therapies. In recent years, interest has grown in the potential of ion channels, particularly transient receptor potential (TRP) ion channels, in cancer progression and treatment resistance. TRP channels, including TRPC6 and TRPM7, have been implicated in a variety of cellular processes that promote tumor growth, survival, and metastasis [13,14]. These channels facilitate calcium influx, which is critical in regulating pathways associated with cell proliferation, migration, and apoptosis. Studies have shown that the overexpression of TRPC6 and TRPM7 correlates with more aggressive cancer phenotypes, as they help cancer cells adapt to the stressful tumor microenvironment by maintaining calcium homeostasis. Specifically, TRPC6 has been found to play a role in enhancing cell migration and invasion, while TRPM7 is known to regulate cell survival mechanisms through its dual ion channel and kinase functions [15,16]. Given their prominent role in cancer biology, TRP channels present an appealing target for developing complementary therapeutic strategies.

In the context of diagnosis and treatment, gastric cancer is typically diagnosed using endoscopy and biopsy samples, followed by determining the histological grade (G) and conducting microbiological analyses [17]. Following diagnosis, imaging techniques are employed for staging, which is then followed by a multidisciplinary discussion to assess treatment options. This process includes considering neoadjuvant FLOT therapy or proceeding directly to surgery [18]. In more advanced cases, bidirectional approaches like Pressurized Intraperitoneal Aerosol Chemotherapy (PIPAC) can be considered. During surgical intervention, subtotal or total gastrectomy with D1 or D2 lymphadenectomy is performed, and in some cases, extended lymphadenectomy may be necessary [19].

There are limited studies on the role of the B7 family of genes in gastric cancer. It was discovered that the expression of *B7H6* gene (also known as *NCR3LG1*; natural killer cell cytotoxicity receptor 3 ligand 1) was elevated in gastric cancer [20]. Prior research has shown that the presence of *B7H6* in cancer cells contributes to immunosuppressive processes by interacting with NKp30 and ILC-2 (type 2 innate lymphoid cells) [21]. This result substantiates a method whereby the production of *B7H6* by cancer cells may increase the survival of tumors. Suppression of *B7H6* mRNA by the use of siRNA in cell lines is highly correlated with a decrease in receptor responsiveness [21]. The lack of *B7H6* in normal tissue, along with its high presence in cancer cells, indicates that its expression might be linked to the prognosis of tumors in a significant number of patients [22,23]. *B7H6* interacts with NKp30, leading to the activation of NK cell cytotoxicity and the release of cytokines. Consequently, *B7H6* functions as an intrinsic molecule inside tumors [24].

Docetaxel, a semisynthetic taxane, is an effective chemotherapy medicine for treating cancer, including gastrointestinal cancer [25]. Docetaxel is a commonly used medication that, due to its suppression of microtubule disintegration via inhibition of microtubule depolymerization, causes resistance that makes therapy ineffective for gastric cancer [26]. However, there is increasing evidence that cancer treatments that combine chemotherapy with siRNA strategies may be a more promising approach for enhancing the effectiveness of anti-cancer medications [27]. Combination therapy is a potent and crucial approach in cancer treatment, including gastric cancer, because of its ability to produce synergistic effects. This therapy modality involves the combination of two or more therapeutic components, resulting in enhanced treatment efficacy [6,28]. In this regard, the objective of this work was to employ siRNA to restrict the expression of the *B7H6* gene while also administering the docetaxel chemotherapeutic medication in order to examine its inhibitory impact on the MKN-45 gastric cancer cell line.

In this study, we focus on the combination of TRP channel modulation with *B7H6* suppression to enhance the chemosensitivity of gastric cancer cells to docetaxel. By integrating gene-silencing techniques targeting *B7H6* with the potential regulatory effects on *TRPC6* and *TRPM7*, we aim to explore a novel approach to overcoming drug resistance in gastric cancer therapy. Understanding the interactions between these ion channels and *B7H6* may reveal additional pathways for targeted treatment.

## 2. Materials and Methods

### 2.1. Cell Culture and Cell Lines Selection

In order to determine an appropriate cell line for the study, MKN45, AGS, and KATO II cell lines were obtained in a frozen state from the Sigma-Aldrich (Saint Louis, MO, USA) and cultivated in RPMI-1640 medium with 1% penicillin and 10% fetal bovine serum (FBS) (all obtained from Sigma-Aldrich) in a humidified incubator (Sanyo, Japan) of 5% CO_2_ at 37 °C. Subsequently, the total RNA isolation was performed by Trizol reagent (RiboEx Kit, GeneAll, Seoul, Republic of Korea) and cDNA synthesis by the Biofact kit (Daejeon, Republic of Korea). By using quantitative real-time PCR (qRT-PCR), the expression levels of *B7H6* gene in the desired cell lines were assessed. The SYBR Premix Ex Taq (Biofact) and Step-One Plus real-time PCR (Applied Biosystems, Thermo Fisher Scientific, Waltham, MA, USA) were also used. The internal monitoring and control used an 18S primer. The mRNA expression levels were evaluated using the 2^−∆∆ct^ method.

### 2.2. Transfection of B7H6 siRNA

In this experiment, two groups of the selected cell line were used: one group of MKN-45 cells that was transfected with *B7H6* siRNA (Santa Cruz, CA, USA) for 24 h and a control group (not transfected cells). Different concentrations of *B7H6* siRNA (40, 60, 80, and 100 pmol) were used to determine the appropriate dose. The cells were initially cultured in a 25 cc flask, and once they reached the 70% confluency, they were detached from the bottom of the flask using Trypsin EDTA (Gibco, Thermo Fisher Scientific, USA). The contents of the flask were then transferred to a 15 mL Falcon tube and centrifuged at 1300 rpm for 10 min. After centrifugation, the supernatant liquid was removed, and the cell pellet was dissolved in 2 mL of transfection buffer (Sigma-Aldrich, USA). To electroporate, 0.4 cuvettes were cooled on ice. Then, 500 µL of buffer containing 12 million cells was added to each cuvette after multiple pipetting of the cell solution. (All stages following transfer to cuvettes were sterile, on ice, and ribonuclease-free.) The Gene Pulser Xcell Transfection Electroporation System (Bio-Rad, Hercules, CA, USA) was used to electroporate with a voltage of 160 and a capacity of 500.

### 2.3. MTT Assay and Cell Viability

To determine the optimum dose of the docetaxel drug (IC50 and IC25) and the investigation of cell viability after transfection with *B7H6* siRNA in combination with the drug and the impact on the proliferation of MKN-45 cells, the MTT assay was used. In order to evaluate the effects of docetaxel on MKN-45 cells, a total of 12 × 10^3^ cells were distributed in 96-well plates using RPMI 1640 culture medium and 10% FBS. After a 24 h period, different concentrations of the drug, ranging from 0.4 to 50 mL of the drug solution, were prepared. After 24 h, the substances in each well were removed, and 150 microliters of MTT solution (Sigma, USA) was added to each cell and left to incubate for a period of 4 h. After removing the medium, 200 μL of dimethyl sulfoxide (DMSO) from Sigma Aldrich was added to each well. This was performed to start the process of dissolving the formazan crystals, which had been incubated for 30 min. Subsequently, the procedure of reading was performed using an ELISA reader (Tecan, Männedorf, Switzerland) with a wavelength of 570/630 nm that recorded the numerical values, which were then analyzed using Prism GraphPad v. 10.4.0 software. Following this procedure, the viability of cells post transfection was assessed using the same methodology, with the exception that half of the cells underwent transfection, while the other half represented the control group of non-transfected cells.

### 2.4. Wound-Healing Assay

In order to assess the migration capability of cells after siRNA transfection, several cell groups were used as in previous experiments. Categories: control group (not transfected), IC50 drug-treated group, IC25 drug-treated group, *B7H6*-transfected group, IC50 drug-treated and *B7H6*-transfected group, and IC25 drug-treated and *B7H6*-transfected group. For this experiment, MKN-45 cells were cultivated, and after they reached the desired confluency, transfection was carried out. Subsequently, 5 × 10^4^ cells were seeded in each well of a 12-well plate (in duplicate). Once the cells achieved a density of 70% and completely covered the bottom of the plate, a scratch was created in each well using a 10 µL pipette tip. Next, the supernatant culture media was removed and rinsed with PBS. Subsequently, 1 mL of the complete culture medium was administered to the control group, while the other wells received the drug-containing culture medium along with siRNA. To evaluate the migration of particles from the edge to the interior of a scratch, the area was studied and photographed using an inverted microscope (Model XDS-3; Optika Microscopes, Bergamo, Italy) at 0 h (test start), 24 h, and 48 h.

### 2.5. Colony-Forming Unit Assay

This experiment aimed to examine the impact of docetaxel and siRNA on the colony formation of MKN-45 cancer cells. The study consisted of the same six groups as the previous experiments. To carry out this procedure, after the cultivation of the cells in a T25 flask, the cells of the required groups were isolated and transfected. Subsequently, a quantity of 3 × 10^3^ cells was seeded into each well of the 6-well plate. After a period of 24 h, the cells belonging to the targeted groups were exposed to dosages of IC25 and IC50. Subsequently, the plate was moved to the incubator to facilitate cell growth and colony expansion. Once the cells reached confluency after 3–4 days, and adequate colonies formed, the supernatant of the cells was removed, and the wells were carefully rinsed with PBS. Next, 1 cc of crystal violet dye was carefully poured into each of the plate wells and left at room temperature for 30 min. Subsequently, the crystal violet dye was carefully collected from the edges of the well using a pipette and then discarded. The well was then rinsed once more with 1 mL of PBS. Ultimately, the colonies in each well were captured in photographs and quantified using an inverted microscope (Model XDS-3; Optika Microscopes, Bergamo, Italy).

### 2.6. Apoptosis Assay

In order to conduct this experiment, the cells were categorized into six distinct groups: the control group, the group treated with docetaxel at the IC50 concentration, the group treated with docetaxel at the IC25 concentration, the group transfected with *B7H6* siRNA, the group transfected with *B7H6* siRNA and treated with docetaxel at the IC50 concentration, and the group transfected *B7H6* siRNA and treated with the IC25 concentration. Cells were trypsinized using Trypsin EDTA, and the MKN-45 transfected and treated cells were transferred to microtubes once they reached optimum confluency after 48 h at 37 °C. They were then washed and centrifuged in two steps at a temperature of 4 °C and a speed of 1200 rpm for 10 min. Then, the supernatant was removed, and after that, the FITC and PI staining kits were applied. The cell pellet was rinsed with Annexin kit buffer and then centrifuged (4 °C, 1200 rpm, 5 min). The pellet was resuspended in 197.5 μL of kit buffer, and 5 μL of FITC labeled with Annexin V was added. Subsequently, it was incubated in the dark at room temperature for 15 min. The cells were then washed again with the kit buffer. Finally, the cells were resuspended in 200 μL of the kit buffer, and 5 μL of PI dye was added. This mixture was incubated for 10 min and analyzed using a flow cytometry device (FACSQuant; Milteny, Bergisch Gladbach, Germany). For data processing, FlowJo software v10.10 (Ashland, OR, USA) was used.

### 2.7. Cell Cycle Analysis

In this experiment, we conducted six test groups following the apoptosis method. The procedure was carried out until the cells were washed, and a cell pellet was obtained. Subsequently, 1 mL of 75% ethanol was added drop by drop to each microtube on a vortex to fix the cells. The microtubes were then maintained at a temperature of −20 °C for 24 h. After this period, the cells were centrifuged for 10 min in a refrigerated centrifuge operating at 4 °C and at a speed of 1200 rpm. The cell pellet was completely resuspended in 500 μL of PBS along with 5 μL of RNase A and then incubated at 37 °C for 30 min. Subsequently, the sample was centrifugated for a duration of 10 min at 4 °C and a speed of 1200 rpm. The cell pellet was dissolved in 1 mL of PBS containing 1 μL of Triton and 1 μL of DAPI. The mixture was then incubated for 10 min at room temperature in the dark. Subsequently, the sample was centrifugated again at the same circumstances. The analysis was then conducted using a flow cytometry device (FACSQuant; Milteny, Germany), and the obtained data were processed with the latest version of FlowJo software v10.10 (Ashland, OR, USA).

### 2.8. RNA Extraction, cDNA Synthesis, and qRT-PCR

MKN-45 cells were seeded in 6-well plates at a concentration of 4 × 10^5^ cells per well, including control and transfected groups. The wells were then treated according to the protocol. The Trizol (RiboEx Kit, GeneAll, Republic of Korea) was used for the extraction of total RNA, and the synthesis of cDNA was carried out following the directions provided by the Biofact kit (Daejeon, Republic of Korea). The gene expression levels of *B7H6*, *BAX*, *BCL-2*, and *Caspase-3* were assessed. SYBR Premix Ex Taq (Biofact, Daejeon, Republic of Korea) and Step-One Plus real-time PCR apparatus from Applied Biosystems, Thermo Fisher Scientific, USA, were used for qRT-PCR analysis of gene expression levels. The primer sequences are presented in Table 1, provided by Bioneer (Daejeon, Republic of Korea). The internal monitoring and control used an 18S primer. The mRNA expression levels were evaluated using the 2^−∆∆ct^ method.

### 2.9. Statistical Analysis

The experiments were replicated three times. The findings were calculated using descriptive statistics and reported as the mean ± standard deviation (mean ± SD). A *p*-value less than 0.05 was considered to be statistically significant. The statistical significance of the data was evaluated using GraphPad Prism v8 (San Diego, CA, USA, www.graphpad.com, accessed on 22 June 2024) by the use of Student’s *t*-test, one-way ANOVA, and two-way ANOVA.

## 3. Results

### 3.1. Cell Line Selection

**Figure 1:**
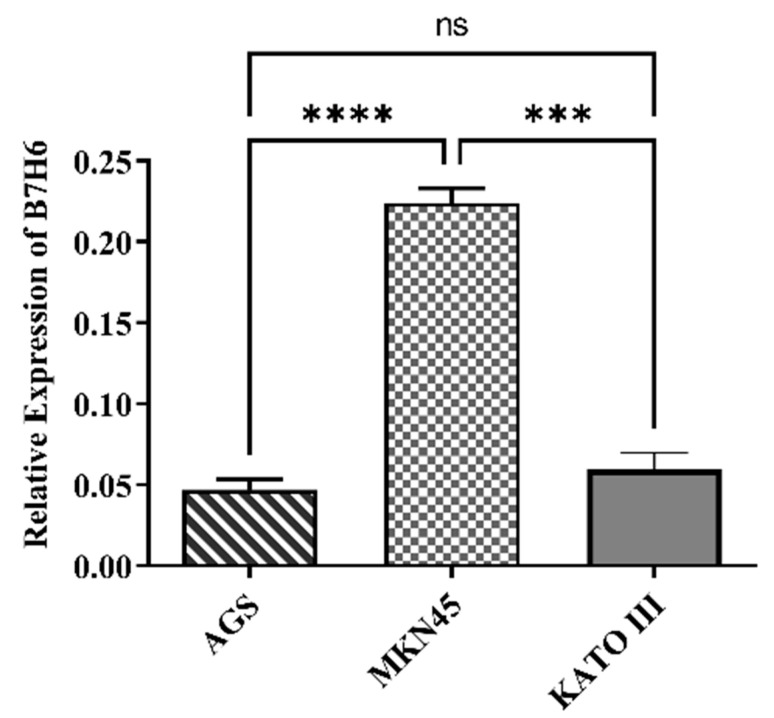
Analysis of the B7H6 immune checkpoint gene expression in gastric cancer cell lines. The MKN-45 cell line has the most significant level of expression (*** *p* < 0.001; **** *p* < 0.0001).

According to the data obtained from the expression level of *B7H6* gene in the gastric cancer cell lines AGS, MKN-45, and KATO III, the MKN-45 cell line was deemed more appropriate (Figure 1). Therefore, this cell line was chosen for the further test procedures.

### 3.2. Optimizing the Dosage of B7H6 siRNA Using the Electroporation Technique

**Figure 2:**
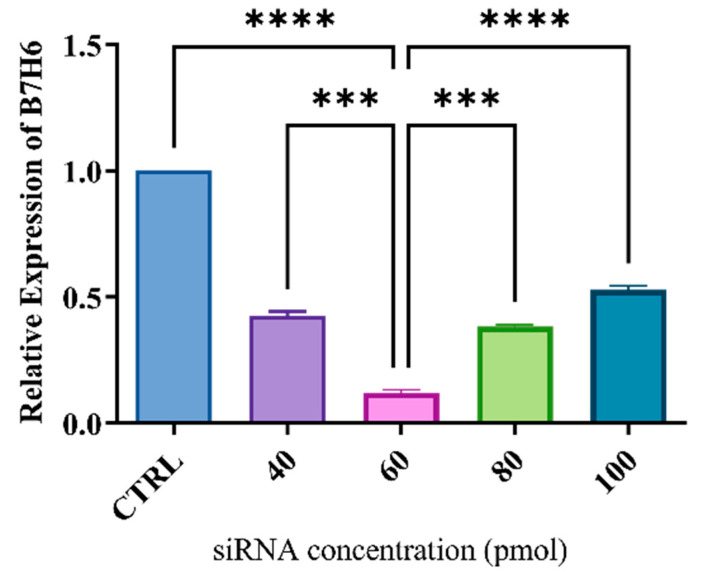
The expression of the *B7H6* gene in MKN-45 cells was inhibited in a dose-dependent manner using particular siRNAs at concentrations of 40, 60, 80, and 100 pmol for 24 h. The data indicate a significant reduction in *B7H6* expression, with the most prominent decline found at a concentration of 60 pmol siRNA (*** *p* < 0.001; **** *p* < 0.0001).

The investigation focused on the inhibition of *B7H6* gene expression in MKN-45 cells using particular siRNAs at dosages of 40, 60, 80, and 100 pmol. The quantitative information of each sample in qRT-PCR was standardized using the expression of the *GAPDH* gene. The findings demonstrated that the transfection of cells with particular siRNA for 24 h significantly reduced the expression of the *B7H6* gene in a way that was dependent on the dosage, as compared to the control group. Based on Figure 2, the findings indicate a substantial reduction in *B7H6* gene expression in MKN-45 cells that were transfected with targeted siRNA of the specified genes at a concentration of 60 pmol.

### 3.3. Assessing Viability of MKN-45 Cells Transfected with B7H6 siRNA and Treated with Docetaxel Alone and in Combination for Potential Synergy

**Figure 3:**
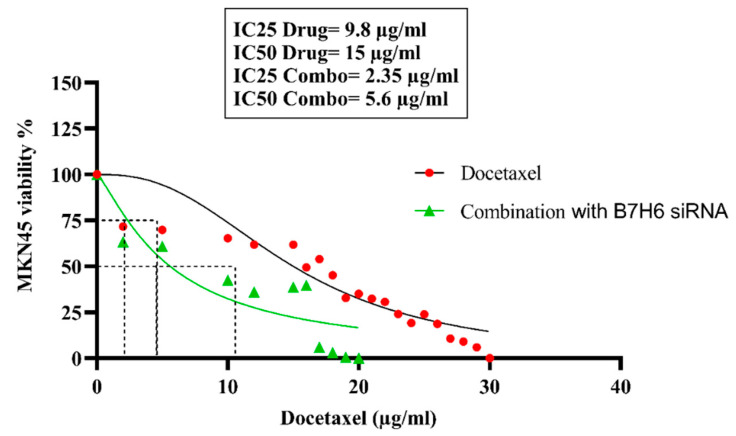
The MTT test findings demonstrate the suppressive effects of docetaxel on the vitality of MKN-45 cells. At concentrations of 15 μg/mL and 9.8 μg/mL, docetaxel decreased cell viability to 50% (IC50) and 25% (IC25), respectively. Following transfection, the IC50 and IC25 values exhibited a drop to 5.6 μg/mL and 2.35 μg/mL, respectively, suggesting an improved responsiveness to docetaxel.

The MTT assay was used for determining the inhibitory concentrations and viability of MKN-45 cells in response to docetaxel. Our results demonstrate that the application of docetaxel at concentrations ranging from 0.4 μg/mL to 40 μg/mL consistently resulted in a reduction in the viability of MKN-45 cells. The treatment of cells with 15 μg/mL and 9.8 μg/mL of docetaxel resulted in a reduction in cell viability to 50% and 25% respectively, compared to untreated cells. Thus, these doses were designated as IC50 and IC25 of docetaxel for use in further evaluations. In addition, the transfected cells were assessed using the MTT assay to evaluate their viability after drug treatment. The findings showed that after transfection, the IC 25 and IC 50 values for docetaxel were dramatically reduced compared to the non-transfected groups. The IC 50 concentration fell by 5.6 μg/mL, while the IC 25 decreased by 2.35 μg/mL (Figure 3).

### 3.4. Investigation of MKN-45 Cell Migration with B7H6 siRNA and Docetaxel Treatment, Individually and Combined, Using Wound-Healing Assay

**Figure 4:**
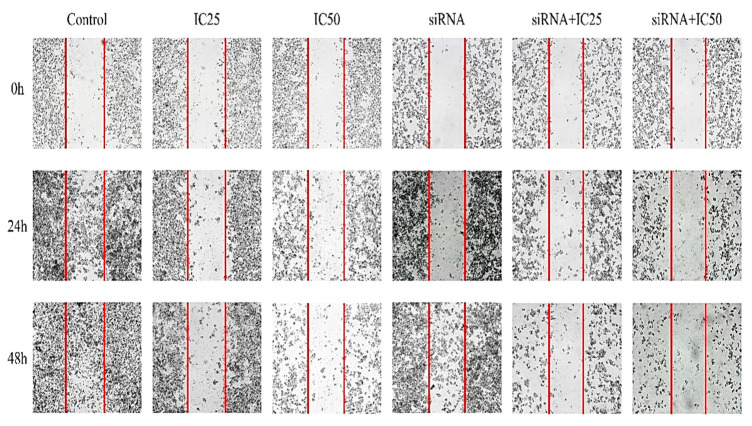
Wound-healing assay showing the impact of *B7H6* siRNA and docetaxel on MKN-45 cell migration at 0, 24, and 48 h. Cells treated with the combination of *B7H6* siRNA and docetaxel, especially at IC50, exhibited significantly reduced migration compared to those treated with either treatment alone.

A wound-healing test was conducted to examine the impact of co-administering *B7H6* siRNA and docetaxel on the migration of MKN-45 cells. The cells were photographed using a microscope at 0, 24, and 48 h following transfection and treatment. The resulting images are shown as a series of comparison pictures in Figure 4. The comparison of the images reveals a considerable reduction in the migratory rate of cells that were simultaneously treated with *B7H6* siRNA and docetaxel. The groups treated individually with *B7H6* siRNA and docetaxel also showed a reduction in migration. However, the decline was much greater and more significant in the combined group, specifically the IC50 combination group.

### 3.5. Combined Treatment with B7H6 siRNA and Docetaxel Results of Colony-Forming Ability in MKN-45 Cells

**Figure 5:**
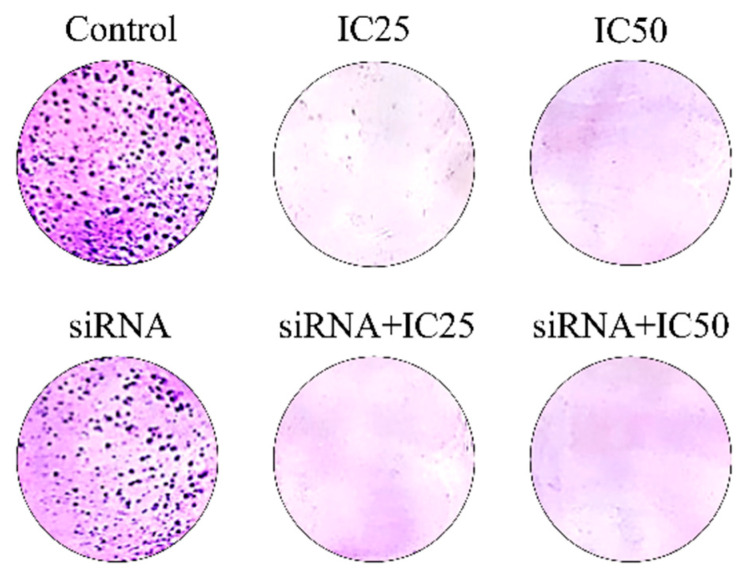
Colony-forming unit assay showing colony formation was reduced with *B7H6* siRNA, further decreased with docetaxel, and nearly eliminated with the combination treatment.

The colony-forming unit assay findings shown in Figure 5 indicate a decrease in colony formation in the group transfected with *B7H6* siRNA compared to the control group. The decrease in colony formation was significant in the cell groups treated with docetaxel alone and much more significant in the group treated with a combination of the drug and *B7H6* siRNA, to the extent that essentially no colony formation was detected in these two groups.

### 3.6. Flow Cytometry Analysis of B7H6 siRNA and Docetaxel-Induced Apoptosis in MKN-45 Cells

**Figure 6:**
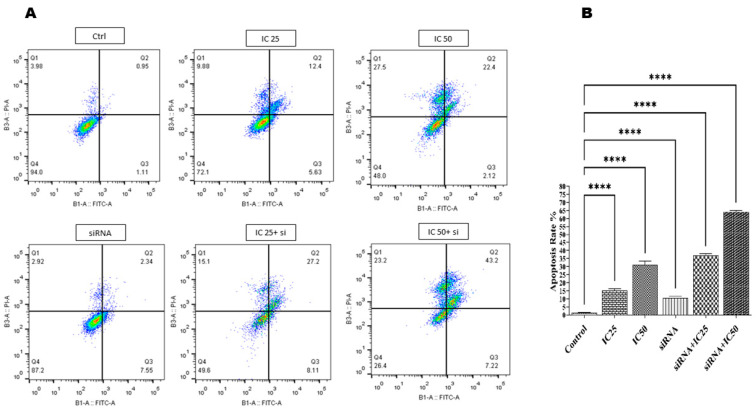
(**A**) Apoptosis in MKN-45 cells treated with docetaxel at IC25 and IC50 doses. When docetaxel was applied alone at IC25 and IC50, the apoptosis rates observed were 13.89% and 31.8%, respectively. This indicates that while docetaxel independently promotes apoptosis, the effect is limited compared to combination treatments. (**B**) Apoptosis in MKN-45 cells treated with a combination of *B7H6* siRNA and docetaxel at IC25 and IC50 doses. The combination treatment significantly enhanced apoptosis rates, reaching 36.4% for the IC25 dose and 63.8% for the IC50 dose. This substantial increase highlights the synergistic effect of *B7H6* siRNA in enhancing the chemosensitivity of MKN-45 cells to docetaxel. Statistical analysis confirmed that these differences were highly significant (**** *p* < 0.0001).

The impact of *B7H6* siRNA used together with docetaxel on progressing the apoptosis in the MKN-45 cell line was evaluated using Annexin V staining. The results from Figure 6A and the analysis in Figure 6B were used to assess the levels of apoptosis induction in the MKN-45 cell line after transfection with *B7H6* siRNA and subsequent treatment with docetaxel compared to the non-transfected groups. The group that was transfected with *B7H6* siRNA alone had an apoptosis rate of 10.94%, which did not show a significant difference compared to the control group. Concurrently, when *B7H6* siRNA was combined with the docetaxel at the IC50 concentration, it resulted in a 63.8% apoptosis rate. This rate was much higher than the apoptosis rate of the drug alone at the same dose, which was 31.8%. Furthermore, the co-administration of the medication at the IC25 concentration together with *B7H6* siRNA resulted in a 36.4% rate of apoptosis. This rate was much higher compared to the treatment of cells with the drug alone at the same dose, which yielded a 13.89% apoptosis rate.

### 3.7. Analysis of Flow Cytometry Reveals the Effects of B7H6 siRNA and Docetaxel Treatment on Cell Cycle in MKN-45 Cells

**Figure 7:**
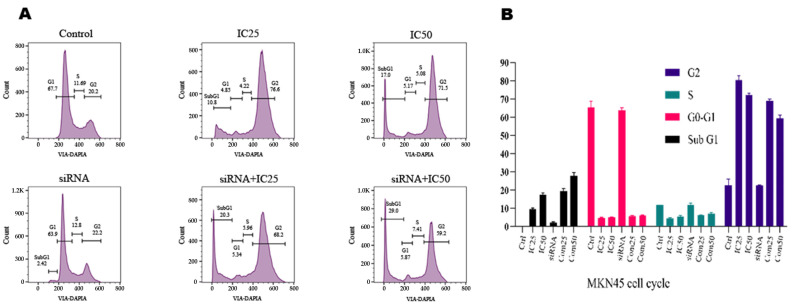
(**A**) Flow cytometry plots for the control group and cells treated with IC25 and IC50 doses of docetaxel. (**B**) Flow cytometry plots for cells treated with siRNA alone and in combination with IC25 and IC50 doses of docetaxel. Flow cytometry analysis of MKN-45 cells treated with docetaxel and *B7H6* siRNA demonstrates significant changes in cell cycle distribution across various treatment groups, including control, IC25, IC50, siRNA alone, and combinations of siRNA with IC25 and IC50 doses of docetaxel. Notably, treatment with docetaxel, especially when combined with *B7H6* siRNA, led to marked increases in sub-G1 (apoptotic) and G2-M phase arrest, indicative of enhanced apoptosis and cell cycle disruption. The combined treatment with IC25 and IC50 doses of docetaxel and siRNA significantly elevated the sub-G1 and G2-M phase cell populations compared to treatment with docetaxel alone.

According to the results obtained from the flow cytometry method to investigate the effect of transfection of *B7H6* siRNA and docetaxel drug on cell cycle arrest, cells treated with doses of docetaxel drug and cells both transfected with *B7H6* siRNA and treated with docetaxel drug in the sub-G1 and G2 phases of cell cycle arrest were observed. Flow cytometry analysis revealed that treatment with docetaxel significantly increased the population of sub-G1 and G2-M phase-arrested cells compared to the control group. In the IC25-treated groups, there was a 10% increase in sub-G1 cells and a 78.6% increase in G2-M cells. In the IC50-treated groups, there was an 18% increase in sub-G1 cells and a 71.5% increase in G2-M cells. Furthermore, MKN-45 cells were treated with a combined therapy, including the administration of docetaxel and *B7H6* siRNA. The findings show that the number of sub-G1 phase cells rose from 2.42 to 18 in the IC25+ siRNA group and from 2.42 to 20.3 in the IC50+ siRNA group compared to the treatment group. The observed alterations in the G2-M phase ranged from 22.2% to 68.2% in the IC25+ siRNA group and from 22.2% to 58.2% in the IC50+ siRNA group (Figure 7).

### 3.8. Expression Changes in Target Genes in MKN-45 Cells Following B7H6 siRNA Transfection and Docetaxel Treatment

**Figure 8:**
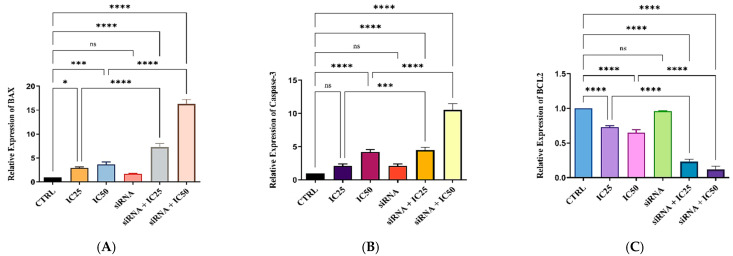
qRT-PCR analysis of apoptosis-related gene expression in MKN-45 cells. The combined treatment with *B7H6* siRNA and docetaxel significantly modulated the expression of key apoptotic markers. *Bax* (**A**) and caspase-3 (**B**) levels were notably increased, indicating enhanced pro-apoptotic activity, while *Bcl-2* expression (**C**) was significantly reduced, suggesting a decrease in anti-apoptotic signaling. These changes compared to individual treatments and control groups highlight the potentiation of apoptosis induced by the combination therapy. Asterisks (*, ***, ****) in the graph represent varying levels of statistical significance, with * indicating *p* < 0.05, *** indicating *p* < 0.001 and **** indicating *p* < 0.0001 reflecting progressively higher levels of confidence in the observed differences.

In order to validate the findings of this research and explore the process by which apoptosis is induced by the treatments, the mRNA expression levels of key regulators of cell death, such as *BAX*, *BCL-2*, and *CASPASE-3*, were assessed using qRT-PCR. Results in Figure 8 demonstrate that the expression of the *BCL-2* pro-survival gene was markedly reduced after the transfection of *B7H6* siRNA and treatment with docetaxel medication in comparison to the control cells. The combination of treatments resulted in the lowest level of *BCL-2* expression (*p* < 0.0001). In addition, the combination treatment of MKN-45 cells with *B7H6* siRNA and docetaxel significantly increased the levels of *BAX* and *CASPASE-3* expression, which are key factors in inducing apoptosis, compared to separate treatments and control cells. Thus, our results indicate that reducing the presence of *B7H6* may enhance the process of programmed cell death in MKN-45 cells treated with docetaxel via influencing the expression of genes associated with apoptosis.

## 4. Discussion

Gastric cancer is a significant therapeutic problem owing to its tendency to be diagnosed at an advanced stage and the resistance of cancer cells to standard treatments. This research investigated the impact of inhibiting *B7H6* on increasing the sensitivity of MKN-45 gastric cancer cells to docetaxel. The results of our study demonstrated that *B7H6*, an immune checkpoint molecule that is responsible for evading the immune system, plays a vital role in the survival and growth of gastric cancer cells. The B7 family was shown to play a key role in immune evasion and tumor growth in earlier research. One new member of this family, *B7H6*, is a promising target for cancer treatment since it is expressed only on tumor cells and not in normal tissues [29,30].

The protein stimulates natural killer (NK) cells and sets off immune system reactions via its interaction with NKp30. Tumors use *B7H6* to establish an immunosuppressive microenvironment, a strategy used by gastric cancer and other malignancies to evade immune monitoring [31]. In this study, MKN-45 cells were selected above other gastric cancer cell lines, including AGS and KATO III, because of their significant *B7H6* gene expression. The expression of *B7H6* was markedly reduced in these cells with the application of siRNA to suppress it, and a dose of 60 pmol was determined to be optimal. *B7H6* siRNA was transfected into MKN-45 cells, which led to a dramatic decrease in *B7H6* expression and an increase in docetaxel sensitivity.

Gene silencing has promise as a method for overcoming drug resistance; this is shown by the fact that *B7H6* suppression lowers docetaxel IC50 and IC25 levels. According to earlier research, suppressing immune checkpoint proteins enhanced chemotherapy responses, which is in line with our current findings. In a study performed by Fiegler et al., monoclonal antibodies were used to analyze tumor cell B7-H6 protein expression and regulation. The researchers observed that HDACi or knockdown of class I histone deacetylases (HDAC2 or HDAC3) decreased *B7H6* protein and mRNA expression. B7-H6 promoter activity and histone acetylation decreased with this downregulation. In lymphoma and liver cancer samples, elevated *B7H6* mRNA levels were linked with *HDAC3* expression. HDACi also reduced *B7H6*, impairing NK cell-mediated immunity. The discovery of a novel tumor *B7H6* regulatory mechanism suggests immunotherapy and HDACi may be effective cancer treatments [32].

The MTT test findings validated the synergistic impact of co-administering *B7H6* siRNA and docetaxel, resulting in a substantial decrease in cell viability compared to the individual treatment with either drug. Our flow cytometry apoptosis experiments showed that *B7H6* siRNA alone did not significantly boost apoptosis. However, when combined with docetaxel at IC 25 and IC50 dosages, the apoptosis rates were 36.4% and 63.8%, respectively. These rates are considerably greater than the rates found while using docetaxel therapy alone. This is consistent with previous research that found an increase in cell death and a decrease in tumor growth when gene-silencing techniques were combined with chemotherapy [33]. The study of the cell cycle revealed that the simultaneous reduction in *B7H6* and administration of docetaxel resulted in a notable arrest in the sub-G1and G2-M phase, which is a clear indication of DNA fragmentation and apoptosis.

Furthermore, the wound-healing experiment revealed a significant suppression of cell migration, indicating that *B7H6* contributes to the metastatic capacity of gastric cancer cells. The decreased ability of colonies to form in the combination group provides further evidence supporting the influence of *B7H6* suppression on the growth and long-term viability of cancer cells. Our research found that the combination treatment caused a decrease in the expression of anti-apoptotic indicators such as *BCL-2* and an increase in the expression of pro-apoptotic markers such as *BAX* and *CASPASE-3* at the molecular level. This indicates that the reason for the improved treatment response is related to the stimulation of intrinsic apoptotic pathways. These findings align with previous studies conducted by Taghavi et al., which showed that blocking *B7H7* enhances the sensitivity of MCF-7 cells to paclitaxel by promoting apoptosis and altering the expression of crucial genes associated with apoptosis. In addition, the combination treatment induced cell cycle arrest and inhibited the formation of colonies in MCF-7 cells [34]. It is worth noting that TRPC6 and TRPM7 ion channels are also known to play roles in gastric cancer progression. Undertaking investigations into the changes in these channels or discussing their interactions within the context of *B7H6* suppression could provide deeper insights into complementary therapeutic approaches.

Recent studies have highlighted the role of transient receptor potential (TRP) ion channels, particularly TRPC6 and TRPM7, in the progression and metastasis of various cancers, including gastric cancer. These ion channels are involved in regulating calcium homeostasis and cell signaling pathways that contribute to tumor cell proliferation, migration, and survival. In gastric cancer, TRPC6 and TRPM7 have been shown to influence oncogenic processes through modulation of intracellular calcium levels, which is critical for tumor progression [35]. Integrating TRPC6 and TRPM7 into the context of *B7H6* suppression could provide new insights into the synergistic effects on chemosensitivity. *B7H6* suppression may affect calcium-dependent pathways regulated by TRP channels, potentially enhancing apoptosis and reducing proliferation in gastric cancer cells. TRPC6, for example, has been associated with promoting cell migration and invasion, processes that are suppressed by *B7H6* downregulation. Similarly, TRPM7’s role in maintaining calcium influx could be inhibited when B7H6 is targeted, potentially augmenting the effects of docetaxel through increased cellular stress and apoptosis [20,36]. Future studies could explore the combined effects of TRP channel inhibition and *B7H6* suppression to enhance therapeutic outcomes in gastric cancer. Investigating whether TRPC6 and TRPM7 influence the apoptotic pathways activated by *B7H6* siRNA and docetaxel treatment may uncover additional mechanisms that could be exploited for targeted therapy.

## 5. Conclusions

By suppressing *B7H6*, MKN-45 gastric cancer cells exhibit much higher sensitivity to docetaxel-induced apoptosis, cell cycle arrest, reduced migratory ability, and lower colony-forming capability. These results suggest that *B7H6* inhibition in combination with docetaxel is a promising treatment approach for gastric cancer, with the potential to overcome drug-resistance mechanisms and improve clinical outcomes. Subsequent research needs to investigate how to take advantage of this approach and evaluate its effectiveness in other cancer models.

## Figures and Tables

**Table 1:** Primer Sequences for Target Genes in qRT-PCR.

Gene	Forward Primer Sequence (5′→3′)	Reverse Primer Sequence (5′→3′)
*B7H6*	CCGGACTGAGTGCTTCTCCT	CCTGTTGCTGTCCTGGTAGT
*BAX*	TGCAGAGGATGATTGCTGAC	GATCAGCTCGGGCACTTTAG
*BCL-2*	CCTGTGGATGACTGAGTACCTGA	GAGACAGCCAGGAGAAATCAAAC
*Caspase-3*	GAACTGGACTGTGGCATTGAG	AGTTTCAGCATGGTTTGTGAGC
*GAPDH*	GTAACCCGTTGAACCCCATT	CCATCCAATCGGTAGTAGCG

## Data Availability

Data are available on request.
